# Efficacy and Safety of Chemotherapy in Elderly Patients with Unresectable Pancreatic Cancer

**DOI:** 10.3390/jcm12093334

**Published:** 2023-05-08

**Authors:** Byung Soo Kwan, Ok Jae Lee, Hyun Jin Kim, Kwang Min Kim, Sang Goon Shim, Dae Hyeon Cho, Sung Min Kong, Jun Young Kim, Jun Ho Ji

**Affiliations:** 1 Department of Medicine, Gyeongsang National University College of Medicine, Jinju 52727, Republic of Korea; 2 Department of Internal Medicine, Samsung Changwon Hospital, Sungkyunkwan University School of Medicine, Changwon 51353, Republic of Korea; 3 Department of Internal Medicine, Gyeongsang National University College of Medicine and Gyeongsang National University Hospital, Jinju 52727, Republic of Korea; 4 Institute of Health Sciences, Gyeongsang National University, Jinju 52727, Republic of Korea; 5 Department of Internal Medicine, Gyeongsang National University College of Medicine and Gyeongsang National University Changwon Hospital, Changwon 51472, Republic of Korea

**Keywords:** pancreatic cancer, chemotherapy, efficacy, safety, elderly

## Abstract

Background/Aims: The incidence of pancreatic cancer (PC) is gradually increasing among elderly individuals, but there are insufficient clinical data on elderly individuals. To determine the efficacy and safety of chemotherapy, we compared the. the outcomes of elderly patients with unresectable PC. Methods: We enrolled patients aged 75 years or older diagnosed with PC from 1 January 2010 to 30 November 2021. Propensity score matching (PSM) was used to reduce the heterogeneity of the study population. For efficacy evaluation, the median overall survival (OS) was estimated for the chemotherapy and nonchemotherapy groups. Chemotherapy tolerability evaluations were also investigated. Results: The study included 115 patients, 47 of whom received chemotherapy and 68 who did not. After PSM, compared with the nonchemotherapy group, the chemotherapy group had more myocardial infarctions (14.6 vs. 0.0%, *p* < 0.001) and chronic obstructive pulmonary disease (4.4 vs. 0.0%, *p* = 0.043). The primary endpoint, median OS, was significantly different in the with vs. without chemotherapy groups (203 vs. 106 days, *p* = 0.013). In the chemotherapy group, 10 patients (21.3%) discontinued treatment due to adverse events. However, there were no reports of death due to severe adverse events. Conclusions: This study demonstrated that chemotherapy improved median OS among elderly patients. These data could support the use of chemotherapy for elderly patients with unresectable PC.

## 1. Introduction

Pancreatic cancer (PC) is the third leading cause of cancer-related death in Western countries and the seventh leading cause of cancer-related death in the world [1,2]. In Korea, PC is the eighth most common cancer; however, the five-year survival rate is only 12.2%, and its incidence is gradually increasing in elderly individuals [3,4,5]. It has been reported that treatment outcomes are worse in elderly patients than in younger patients [6,7]. However, elderly patients are underrepresented in existing clinical studies, so data regarding the treatment frequency and intensity for elderly PC patients are generally limited [8,9]. This lack of data presents many limitations in terms of constructing a treatment strategy for elderly individuals. Therefore, data on the treatment response of elderly PC patients are needed.

The possibility of surgery is a very important factor in the prognosis of PC. Depending on the possibility of surgery, the prognosis is markedly different; R0 resection has an average survival of 31.8 months; however, if untreated, life expectancy is only a few months [6,10]. To improve the poor prognosis, chemotherapy is the main treatment for unresectable PC, and recent studies on chemotherapy for PC show promising statistics in terms of improving the survival rate. With the introduction of a combination regimen of 5-FU, leucovorin, irinotecan, and oxaliplatin (FOLFIRINOX) in 2011, the median overall survival time improved to 11.1 months compared to 6.8 months for gemcitabine monotherapy, which has been the standard treatment since 1997 [11,12]. In addition, the nab-paclitaxel plus gemcitabine combined chemotherapy regimen introduced in 2013 was reported to yield a median OS of 8.5 months, a significant improvement in survival compared to the 6.7 months for gemcitabine monotherapy [13].

Recently, we have entered an aging society, and in the past, those aged 65 and over were defined as elderly, 7% or more of the population as an aging society, 14% or more of the population as an aged society, and 20% or more as a super aging society [14]. As the aging society progressed, the elderly also started to be classified according to age. Several previous studies have classified elderly adults between the ages of 65 and 74 years as youngest-old, those between ages 75 and 84 years as middle-old, and those aged over 85 years as oldest-old [15]. The incidence of PC is increasing among elderly individuals, and in recent representative studies, such as the FOLFIRINOX chemotherapy study, patients were an average of 61 years old and in good general condition [12]. Likewise, a previous study that introduced the use of nab-paclitaxel plus gemcitabine studied patients with an average age of 62 years and in good general condition [13]. Since then, many studies have been published, but elderly patients (older than 75 years of age) have been excluded from many studies; although age is an important factor in treatment determination, studies on elderly patients have small sample sizes, and there are few data, especially for the Asian population [16,17,18,19,20].

Therefore, the efficacy and safety of chemotherapy were evaluated in elderly patients older than 75 years of age with unresectable PC in this study. Additionally, the factors for better outcomes were analyzed.

## 2. Materials and Methods

### 2.1. Study Design and Study Population

This study was a single-center retrospective observational case-control study to evaluate the efficacy and safety of chemotherapy in elderly patients with unresectable PC. Enrolled patients were consecutively selected according to inclusion and exclusion criteria. Among the patients with PC diagnosed at Samsung Changwon Hospital, Sungkyunkwan University School of Medicine between 1 January 2010 and 30 November 2021, patients with resectable and borderline-resectable pancreatic cancer (BRPC) and patients lost to follow-up after diagnosis were excluded. In addition, as an Eastern Cooperative Oncology Group (ECOG) performance status of 3 or higher may affect overall survival, it was excluded from the analysis in order to compare only the chemotherapy effect. Patients with unresectable PC, including locally advanced pancreatic cancer (LAPC) and metastatic PC, who were followed were included. The patients were classified into two groups: chemotherapy and nonchemotherapy groups.

Patients who were diagnosed with PC by histological and radiological diagnostic criteria and were assigned an appropriate code for PC according to the International Classification of Disease, tenth revision (ICD-10) were included. The ICD is an internationally standardized disease code, and the ICD-10 codes for PC are C25.0, C25.1, C25.2, C25.3, C25.4, and C25.9.

The resectability of PC was assessed according to the International Study Group for Pancreatic Surgery (ISGPS) guidelines of 2014 and international consensus on the definition and criteria of borderline resectable pancreatic ductal adenocarcinoma of 2017 [21,22]. The evaluation of resectability was based on computed tomography (CT) using a pancreatic protocol. Patients with PC were classified into resectable, BRPC, or unresectable groups according to the degree of invasion of the superior mesenteric vein (SMV) or portal vein (PV) and the common hepatic artery (CHA), superior mesenteric artery (SMA), and celiac artery (CA). Vascular invasion was determined by the degree of fat preservation between the blood vessel and the tumor, the deformation of the vessel shape, stenosis, and occlusion. LAPC and metastatic PC were considered unresectable cancers. LAPC was defined as PC with unreconstructible SMV/portal occlusion, SMA or IVC encasement greater than 180°, or any celiac abutment. Metastatic PC was defined as PC with the presence of distant metastases, including macroscopic para-aortic lymph node (LN) and extra-abdominal LN metastasis.

### 2.2. Data Collection

The authors collected the patients’ demographic data and clinical variables, including body mass index (BMI); Charlson comorbidity index (CCI) score; ECOG performance status; clinical details of pancreatic cancer; regimen, dose adjustment, response, and adverse effects of first line chemotherapy; survival period; and history of surgery or biliary drainage, and then compared these variables between the two groups. Descriptive statistics were used to represent the baseline characteristics of the patients.

Chemotherapy response was assessed every 2–3 months using CT, and it was graded as complete response (CR), partial response (PR), stable disease (SD), or progressive disease (PD) according to the Response Evaluation Criteria in Solid Tumors (RECIST), version 1.1 [23].

Additionally, to evaluate comorbidities, the CCI score was applied and retrospectively investigated through electronic chart review. The CCI details included age, myocardial infarction (MI), congestive heart failure (CHF), peripheral vascular disease, cerebrovascular accident (CVA) or transient ischemic attack, dementia, chronic obstructive pulmonary disease (COPD), connective tissue disease, peptic ulcer disease (PUD), liver disease, diabetes mellitus (DM), hemiplegia, severity of chronic kidney disease (CKD), solid tumor, leukemia, lymphoma, and acquired immune deficiency syndrome [24]. The functional status of cancer patients was investigated based on the widely used ECOG performance status. ECOG performance status is one of the standard criteria for measuring the effect of disease on a patient’s ability to perform daily activities and is divided into stages ranging from 0, which is the standard for normal daily life, to 5, meaning death [25].

### 2.3. Outcomes

For chemotherapy efficacy evaluation, the median OS of all patients with or without chemotherapy was estimated as the primary endpoint. The median OS was defined as the length of time from either the date of diagnosis or the start of treatment for PC to death, and half of the patients were still alive at the end of the follow-up period.

The grade and type of toxicities were investigated in all patients who received chemotherapy, including patients for whom treatment was discontinued due to toxicities of chemotherapy. Toxicity was graded according to the National Cancer Institute’s Common Terminology Criteria for Adverse Event (NCI-CTCAE version 4.03) [26].

### 2.4. Statistical Analysis

Heterogeneity was observed in the clinical features of the population groups in this study. There were statistically significant differences in age, BMI, and ECOG performance status between the chemotherapy and nonchemotherapy groups. Therefore, propensity score matching (PSM) was performed to minimize the effects of these differences in the baseline characteristics of the two groups and to reduce potential confounders using a Cox proportional hazards model with adjustment for the following: age, sex, BMI, CCI score, and ECOG performance status. The authors assessed each propensity score matching of the two groups in a 1:2 ratio using Mahalanobis matching; the caliper was set to 0.1 and the predicted probability of the patient with chemotherapy versus the patient without chemotherapy was calculated among all of the patients with PC (n = 115).

Categorical variables are represented as counts and percentages and were compared using Fisher’s exact test or Pearson’s chi-square test. Continuous variables are represented as medians with standard ranges and were compared using Student’s *t* test. The overall survival rate with and without chemotherapy was calculated using the Kaplan–Meier method, and log-rank tests were used to assess differences. Univariate and multivariate analyses were performed using the Cox proportional hazards model. All of the analyses were conducted using Stata version 15.1 (Stata Corporation, College Station, TX, USA). For all analyses, *p* values < 0.05 were considered statistically significant.

### 2.5. Ethics

This study was approved by the Institutional Review Board of Samsung Changwon Hospital, Sungkyunkwan University School of Medicine, the requirement for informed consent was waived (SCMC 2021-12-005), and all procedures were performed according to the guidelines of the Declaration of Helsinki. The researcher could not identify individuals by their data because all data were deidentified. Therefore, inclusion in the study did not jeopardize confidentiality.

## 3. Results

### 3.1. Patient Baseline Characteristics before and after PSM

**Figure 1:**
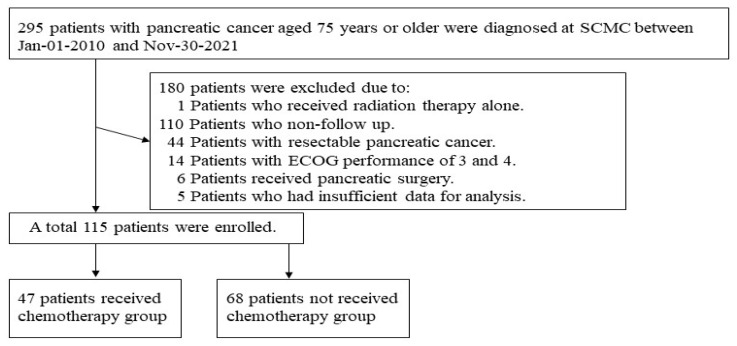
Schematic flow diagram of the selection and disposition of patients. SCMC, Samsung Changwon Hospital, Sungkyunkwan University School of Medicine.

A total of 295 patients aged 75 years or older were diagnosed with PC at SCMC during the study period. Among them, 180 patients were excluded, and 115 were selected based on the inclusion and exclusion criteria (Figure 1). Among the 115 patients, 47 received chemotherapy and 68 did not receive chemotherapy. In the group that did not receive chemotherapy, 22.1% of patients had worsened underlying diseases, 5.9% were unsure of the cause, and 72.1% of patients refused treatment out of fear of chemotherapy. The baseline characteristics are described in Table 1 and Table 2. The study included 51 males and 64 females, and the mean age was 83.4 ± 5.1 years. ECOG performance status was distributed as follows: 0 points, 27.8%; 1 point, 43.5%; and 2 points, 28.7%. Pancreatic head and uncinate process cancer was diagnosed in 55.7% of cases, LAPC in 18.3% of cases, and metastatic PC in 81.7% of cases. Distant metastasis was evaluated via positron emission tomography−computed tomography (PET-CT) in 53.9% of patients and CT without PET-CT in the rest of the patients. Except for four patients who could not be identified by electronic records, 74.5% of all the patients in the chemotherapy group underwent histological testing through EUS-FNA. However, 33.8% of the patients who did not receive chemotherapy did not receive a biopsy.

In the group receiving chemotherapy, the most frequently used first-line chemotherapy was nab-paclitaxel plus gemcitabine (46.8%), and the second most commonly used first-line chemotherapy was gemcitabine monotherapy (34.0%). In the first-line chemotherapy stage, 36.2% of patients underwent dose adjustment, and stable disease (SD) was the most common response type (51.1%) at the first evaluation. Among the patients who received chemotherapy, 17.0% received second-line chemotherapy, 6.4% received third-line chemotherapy, and the average chemotherapy cycle was 6.8 ± 6.4 cycles. The most frequently used second-line chemotherapy was TS-1, a combination oral chemotherapy drug consisting of Tegafur, Gimeracil, and Oteracil monotherapy (50.0%). Twelve patients (23.4%) did not have chemotherapy response evaluations; among them, 10 patients discontinued chemotherapy, and 1 patient did not undergo imaging testing for response evaluation.

Compared with the nonchemotherapy group, the chemotherapy group was younger (80.9 ± 3.9 vs. 85.1 ± 5.1 years, *p* < 0.001), had mostly ECOG performance status values of 0 (44.7 vs. 16.2%, *p* = 0.001), and had more patients with body and tail PC (55.3 vs. 44.7%, *p* = 0.049).

After PSM, a total of 136 patients were analyzed; 45 patients received chemotherapy and 91 patients did not receive chemotherapy. The baseline characteristics are described in Table 3. The study group included 70 males and 66 females, and the mean age was 81.0 ± 4.0 years. In terms of comorbid diseases, compared with the nonchemotherapy group, the chemotherapy group had more MIs (15.6 vs. 0.0%, *p* < 0.001) and COPDs (4.4 vs. 0.0%, *p* = 0.043).

### 3.2. Efficacy of Chemotherapy and Factors Affecting Survival in Elderly Patients with Unresectable PC after PSM

**Figure 2:**
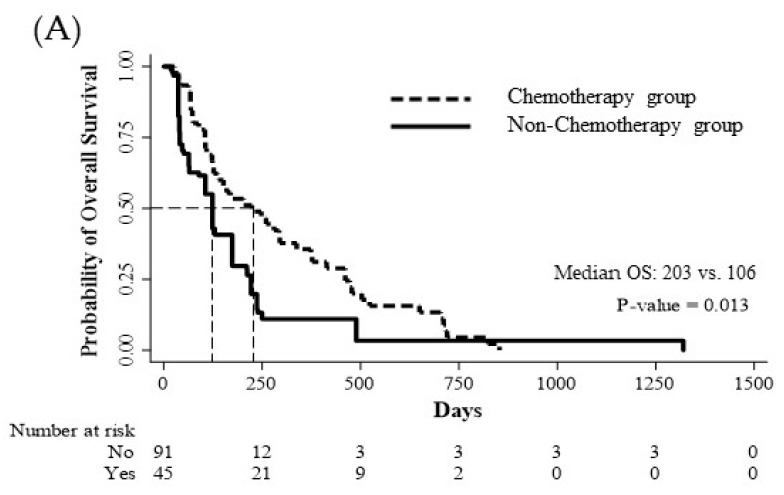

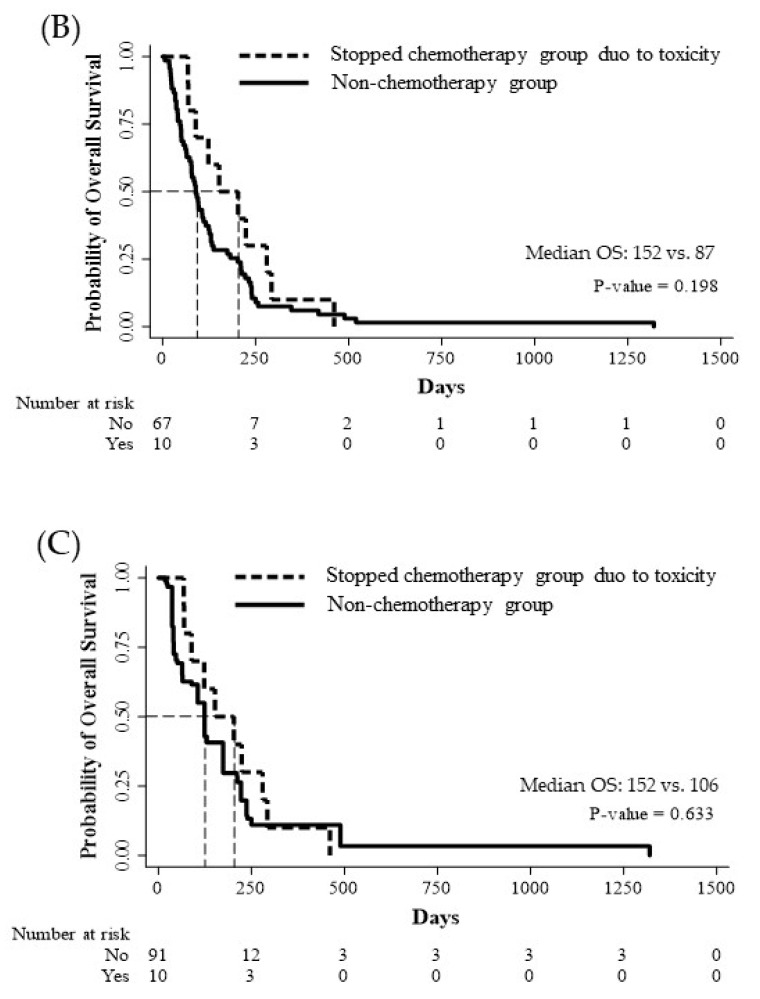
(**A**) Kaplan–Meier curves for overall survival after propensity score matching. Median overall survival was significantly longer in the group receiving chemotherapy than in the group not receiving chemotherapy on the log-rank test (203 vs. 106 days, *p* = 0.013); (**B**) Kaplan–Meier curves for overall survival before propensity score matching. There was no statistically significant difference in the median OS between the group who stopped chemotherapy due to toxicity and the group who did not receive chemotherapy (152 vs. 87 days, *p* = 0.198). (**C**) Kaplan–Meier curves for overall survival after propensity score matching. There was no statistically significant difference in the median OS between the group who stopped chemotherapy due to toxicity and the group who did not receive chemotherapy according to the log-rank test (152 vs. 106 days, *p* = 0.633).

The primary endpoint of this study, OS, was compared between elderly patients with or without chemotherapy (Figure 2). The median OS was significantly different (203 vs. 106 days, *p* = 0.013).

Table 4 shows the results of univariate and multivariate analyses for the factors affecting OS in elderly patients with unresectable PC after PSM. According to the multivariate analysis, chemotherapy (HR, 0.60 (0.41–0.90), *p* = 0.012), BMI (HR, 1.12 (1.05–1.20), *p* = 0.001), ECOG performance status values of 2 (HR, 2.34 (1.21–4.52), *p* = 0.012), and metastatic PC (HR, 1.73 (1.01–2.96), *p* = 0.046) were significantly associated with mortality risk in elderly patients with unresectable PC.

### 3.3. Safety of Chemotherapy in Elderly Patients with Unresectable PC after PSM

Adverse events in the chemotherapy groups are described in Table 5. Common mild adverse events (grades 1 and 2) were fatigue (46.8%) and anorexia (17.0%), while the most common serious adverse events (grades 3 or 4) were fatigue (12.8%), diarrhea (4.3%), and vomiting (2.1%).

In the group who received chemotherapy, a total of 10 patients (21.3%) discontinued chemotherapy due to adverse events: severe fatigue (6), mild fatigue (2), fatigue and febrile neutropenia (1), and fatigue and severe diarrhea (1). According to the chemotherapy regimen, four patients in the gemcitabine monotherapy group, five in the nab-paclitaxel plus gemcitabine combination treatment, and one in the TS-1 monotherapy group stopped treatment during the first chemotherapy cycle. However, there were no reports of death due to severe adverse events.

The median OS was compared between the chemotherapy group with adverse event-related discontinuance and the nonchemotherapy group, but there was no statistically significant difference (152 vs. 87 days, *p* = 0.198). PSM did not reveal a statistically significant difference (152 vs. 106 days, *p* = 0.633) (Figure 2).

## 4. Discussion

In this study, we found that the median OS was significantly improved in elderly patients aged 75 years or older with unresectable PC who received chemotherapy. In addition, there were no deaths due to adverse reactions, and chemotherapy did not have fatal adverse events in patients over 75 years of age.

PC affects many elderly patients. In Western countries, the average age of patients with PC at diagnosis is 72 years, and more than 68% of patients are 65 years or older at diagnosis [5]. Mizrahi et al. [16] reported that the use of a modified dose of FOLFIRINOX in 24 patients with advanced PC over 75 years of age appeared to maintain toxicity and efficacy profiles similar to those in younger patients. Furthermore, a paper on gemcitabine monotherapy targeting the elderly, those over 75 years of age, reported that it is sufficiently effective and tolerable, even in elderly individuals. A recently published paper on nab-paclitaxel plus gemcitabine combination therapy also reported that it is tolerable; although it does not demonstrate its superiority over monotherapy, it was found to have a positive effect on the OS in multivariate analysis [27,28]. In this study, chemotherapy, including a modified dose of FOLFIRINOX, was sufficiently effective for unresectable PC in patients aged 75 years or older, and the median OS was not significantly different from that in the nonchemotherapy group, even when chemotherapy was discontinued due to adverse events.

In addition, the authors identified good prognostic factors affecting the median OS of participants in the multivariate analysis in this study; receiving chemotherapy, lower BMI, lower ECOG performance status value, and nonmetastatic PC were identified as good prognostic factors. In fact, distant metastases, a high value of ECOG performance, and a high BMI are well-known prognostic factors related to pancreatic cancer [29,30,31]. These results are similar to the results of the present study.

The authors identified that chemotherapy was relatively tolerable in patients aged 75 years or older with unresectable PC and did not cause any deaths, and only 21.3% of patients discontinued treatment due to chemotherapy-related adverse events. However, Li et al. [18] reported that the severity or grade of chemotherapy-related toxicities was much higher in older patients. FOLFIRINOX was the main registered treatment in Li et al.’s [18] study, whereas nab-paclitaxel plus gemcitabine combination therapy was the main registered treatment in this study. In fact, FOLFIRINOX led to a higher rate of severe adverse events, including neutropenia, than gemcitabine-based drugs in previous domestic studies, so the difference in the results obtained in the study by Li et al. [18] and the present studies can be explained by the different chemotherapy regimens registered in the studies [32].

Ulrich et al. [33] reported that older patients undergoing chemotherapy experience similar adverse events to younger patients, but may experience stronger adverse events due to underlying health conditions, bone marrow changes, and other physical changes. As adverse events tend to be more pronounced in elderly patients, particularly in terms of hematologic toxicity, the chemotherapy dosage was decreased to improve tolerability [34]. In our study, 10 (21.3%) patients who received chemotherapy experienced severe adverse events, which is lower than the results of other similar studies [12,13,16]. We believe that the lower frequency of severe adverse events in this study was caused by chemotherapy with an adjustable dosage in approximately 36.2% of patients. In addition, approximately 21.3% of patients discontinued chemotherapy, and the discontinuation rate was comparable to that of other studies. In other studies, there was no significant difference in the frequency of adverse events and dose adjustment during chemotherapy between young and elderly patients in terms of chemotherapy dosage adjustment. However, permanent chemotherapy discontinuation due to adverse events was much higher in elderly individuals [35].

In previous studies, younger patients received chemotherapy more often than patients over 70 years of age. It has also been shown that relatively older people tend to avoid chemotherapy because they are afraid of adverse effects [5]. Similarly, in our study, 72.1% of patients who did not receive chemotherapy refused treatment because they were afraid of chemotherapy, which is similar to the results of previous studies. Although there is a lack of studies, it has been found that median OS does not differ by chemotherapy among elderly patients versus nonelderly patients [16]. The results of this study showed that chemotherapy was effective enough for unresectable PC in patients over 75 years of age, and no adverse events leading to death were observed.

## 5. Limitations

This study has limitations, as it is a single-center small-size retrospective study. In addition, during the study period, standard chemotherapy for unresectable pancreatic cancer advanced from gemcitabine monotherapy to nab-paclitaxel plus gemcitabine combination therapy and FOLFIRINOX. However, gemcitabine monotherapy is still maintained as the standard chemotherapy. The majority of patients enrolled in our study received standard chemotherapy at the time. Although this study has limitations as it is not comparing single chemotherapy, it considers the perspective of efficacy and safety when standard chemotherapy is performed in elderly individuals. Therefore, we believe it provides valuable and scarce data, despite its limitations.

## 6. Conclusions

This study demonstrated that chemotherapy improved median OS among elderly patients. In addition, even when chemotherapy was discontinued due to adverse events, median OS was not significantly different from that of the group that did not receive chemotherapy. The number of elderly unresectable PC patients is gradually increasing and expectations for therapeutic effects are rising. The positive results obtained in the present study on chemotherapy and its adverse events among elderly unresectable PC patients will have a good influence on the establishment of evidence-based treatment for elderly patients. Considering these results, the authors suggest that it is better to actively try chemotherapy if chemotherapy is available, rather than to avoid chemotherapy out of fear of severe adverse events, not only among younger patients, but also for elderly patients with unresectable PC.

## Figures and Tables

**Table 1:** Baseline characteristics of patients with unresectable pancreatic cancer before propensity score matching.

	Total Patient (n = 115)	Chemotherapy		*p* Value
		Yes (n = 47)	No (n = 68)	
Age, years	83.4 ± 5.1	80.9 ± 3.9	85.1 ± 5.1	<0.001
Sex, Female	64 (55.7)	22 (46.9)	42 (61.8)	0.112
BMI, kg/m^2^	21.3 ± 3.0	21.9 ± 3.0	20.9 ± 3.0	0.087
Charlson comorbidity index	9.7 ± 1.6	9.5 ± 1.9	9.8 ± 1.4	0.312
MI	10 (8.7)	7 (14.9)	3 (4.4)	0.088
CHF	2 (1.7)	1 (2.1)	1 (1.5)	>0.999
CVA	9 (7.8)	5 (10.6)	4 (5.9)	0.483
COPD	3 (2.6)	2 (4.3)	1 (1.5)	0.566
Connective tissue disease	3 (2.6)	1 (2.1)	2 (2.9)	>0.999
PUD	1 (0.9)	1 (2.1)	0 (0.0)	0.409
Liver disease, severe	2 (1.7)	1 (2.1)	1 (1.5)	>0.999
DM				0.158
Uncomplicated	15 (13.0)	9 (19.2)	6 (8.8)	
End-organ damage	1 (0.9)	0 (0.0)	1 (1.5)	
CKD	2 (1.7)	1 (2.1)	1 (1.5)	>0.999
ECOG performance				0.001
0	32 (27.8)	21 (44.7)	11 (16.2)	
1	50 (43.5)	19 (40.4)	31 (45.6)	
2	33 (28.7)	7 (14.9)	26 (38.2)	
Biliary drainage, yes	53 (46.1)	19 (40.4)	34 (50.0)	0.311
Location of pancreatic cancer				0.049
Head or uncinate	64 (55.7)	21 (44.7)	43 (63.2)	
Body or tail	51 (44.3)	26 (55.3)	25 (36.8)	
Resectability of pancreatic cancer				0.486
Locally advanced	21 (18.3)	10 (21.3)	11 (16.2)	
Metastatic	94 (81.7)	37 (78.7)	57 (83.8)	
PET-CT	62 (53.9)	35 (74.5)	27 (39.7)	<0.001
Histologic diagnosis				<0.001
None	23 (20.0)	0 (0.0)	23 (33.8)	
EUS-FNA	56 (48.7)	35 (74.5)	21 (30.9)	
ERCP & Endoscopic route *	15 (13.0)	3 (6.4)	12 (17.7)	
Percutaneous route **	16 (13.9)	5 (10.6)	11 (16.2)	
Unknown	5 (4.4)	4 (8.5)	1 (1.5)	
Median Overall survival	124 (65–250)	224 (105–461)	89 (47–202)	<0.001

Values are presented median ± ranges or n (%); N, number of patients; %, percentage; BMI, body mass index; MI, myocardial infarction; CHF, congestive heart failure; CVA, cerebrovascular accident; COPD, chronic obstructive pulmonary disease; PUD, peptic ulcer disease; DM, diabetes mellitus; CKD, chronic kidney disease; ECOG, Eastern Cooperative Oncology Group; PET-CT, positron emission tomography−computed tomography; EUS-FNA, endoscopic ultrasound guided fine needle aspiration; ERCP, endoscopic retrograde cholangiopancreatography; * Endoscopic biopsy of the direct invasion of other organs of primary pancreatic cancer; ** Percutaneous biopsy of direct invasion of other organs and metastatic lesions of primary pancreatic cancer.

**Table 2:** Details of the chemotherapy group with unresectable pancreatic cancer (n = 47).

	No. of Patients (%)
Firstline Chemotherapy, regimen	
5-FU	1 (2.1)
Etoposide + cisplatin	1 (2.1)
FOLFIRINOX	4 (8.5)
Gemcitabine	16 (34.0)
Gemcitabine + Cisplatin	1 (2.1)
Gemcitabine + Nab-paclitaxel	22 (46.8)
TS-1	2 (4.3)
Firstline Chemotherapy, dose	
Full dose	30 (63.8)
Adjust dose	17 (36.2)
Firstline Chemotherapy, response *	
PR	10 (21.3)
SD	24 (51.1)
PD	2 (4.3)
Not assessed **	11 (23.4)
Received chemotherapy	
First-line	39 (83.0)
Second-line	5 (10.6)
Third-line	3 (6.4)
Second-line chemotherapy, regimen	
FOLFIRINOX	1 (12.5)
Gemcitabine	1 (12.5)
Gemcitabine + Erlotinib	1 (12.5)
Capecitabine	1 (12.5)
TS-1	4 (50.0)
Mean chemotherapy cycle	6.8 ± 6.4

BMI, body mass index; ECOG, Eastern Cooperative Oncology Group; 5-FU, 5-Fluorouracil; FOLFIRINOX, combination regimen of 5-fluorouracil, leucovorin, irinotecan, and oxaliplatin; TS-1, Tegafur, Gimeracil, and Oteracil combined oral chemotherapy drug; PR, partial response; SD, stable disease; PD, Progression disease; * Chemotherapy response was assessed every 2–3 months using CT and graded according to the Response Evaluation Criteria in Solid Tumors, version 1.1. ** 11 patients were not assessed for chemotherapy response because 10 discontinued chemotherapy and 1 did not have imaging tests for response evaluation.

**Table 3:** Propensity score matched baseline characteristics of the patients with unresectable pancreatic cancer.

	Total Patient (n = 136)	Chemotherapy		*p* Value
		Yes (n = 45)	No (n = 91)	
Age, years	81.0 ± 4.1	80.8 ± 3.6	81.1 ± 4.3	0.698
Sex, Female	66 (48.5)	21 (46.7)	45 (49.5)	0.760
BMI, kg/m^2^	21.6 ± 3.1	21.8 ± 3.0	21.6 ± 3.2	0.648
Charlson comorbidity index	9.4 ± 1.6	9.4 ± 1.9	9.3 ± 1.4	0.634
MI	7 (5.2)	7 (15.6)	0 (0.0)	<0.001
CHF	1 (0.7)	1 (2.2)	0 (0.0)	0.331
CVA	12 (8.8)	5 (11.1)	7 (7.7)	0.531
COPD	2 (1.5)	2 (4.4)	0 (0.0)	0.043
Connective tissue disease	1 (0.7)	1 (2.2)	0 (0.0)	0.153
PUD	1 (0.7)	1 (2.2)	0 (0.0)	0.153
Liver disease, severe	1 (0.7)	1 (2.2)	0 (0.0)	0.153
DM, uncomplicated	19 (14.0)	9 (20.0)	10 (11.0)	0.190
CKD	4 (2.9)	0 (0.0)	4 (4.4)	0.153
ECOG performance				0.390
0	64 (47.1)	20 (44.4)	44 (48.3)	
1	58 (42.6)	18 (40.0)	40 (44.0)	
2	14 (10.3)	7 (15.6)	7 (7.7)	
Biliary drainage, yes	59 (43.4)	18 (40.0)	41 (45.1)	0.576
Location of pancreatic cancer				0.078
Head or uncinate	75 (55.2)	20 (44.4)	55 (60.4)	
Body or tail	61 (44.8)	25 (55.6)	36 (39.6)	
Resectability of pancreatic cancer				0.051
Locally advanced	19 (14.0)	10 (22.2)	9 (9.9)	
Metastatic	117 (86.0)	35 (77.8)	82 (90.1)	
PET-CT	47 (34.6)	11 (24.4)	36 (39.6)	0.081
Histologic diagnosis				<0.001
None	16 (11.8)	0 (0.0)	16 (17.6)	
EUS-FNA	81 (59.6)	35 (77.8)	46 (50.6)	
ERCP & Endoscopic route *	13 (9.6)	3 (6.7)	10 (11.0)	
Percutaneous route **	23 (16.9)	4 (8.9)	19 (20.9)	
Unknown	3 (2.2)	3 (6.7)	0 (0.0)	
Median Overall survival	126 (64–244)	224 (105–461)	124 (41–222)	0.001

Values are presented as the median ± range or n (%); N, number of patients; %, percentage; BMI, body mass index; MI, myocardial infarction; CHF, congestive heart failure; CVA, cerebrovascular accident; COPD, chronic obstructive pulmonary disease; PUD, peptic ulcer disease; DM, diabetes mellitus; CKD, chronic kidney disease; ECOG, Eastern Cooperative Oncology Group; PET-CT, positron emission tomography−computed tomography; EUS-FNA, endoscopic ultrasound-guided fine needle aspiration; ERCP, endoscopic retrograde cholangiopancreatography; * Endoscopic biopsy of the direct invasion of other organs of primary pancreatic cancer; ** Percutaneous biopsy of direct invasion of other organs and metastatic lesions of primary pancreatic cancer.

**Table 4:** Univariate and multivariate analyses for the factors of mortality in patients with unresectable pancreatic cancer after propensity score matching.

	Univariate		Multivariate	
	HR (95% CI)	*p* Value	HR (95% CI)	*p* Value
Chemotherapy				
Yes	0.64 (0.44–0.92)	0.017	0.60 (0.41–0.90)	0.012
BMI	1.10 (1.03–1.18)	0.003	1.12 (1.05–1.20)	0.001
Charlson comorbidity index	1.13 (1.00–1.27)	0.056		
ECOG performance				
0	Reference		Reference	
1	1.51 (1.05–2.18)	0.028	1.42 (0.97–2.08)	0.070
2	2.61 (1.44–4.73)	0.001	2.34 (1.21–4.52)	0.012
Biliary drainage, yes	1.02 (0.72–1.45)	0.892		
Location of pancreatic cancer				
Head or uncinate	reference			
Body or tail	0.91 (0.64–1.28)	0.585		
Resectability of pancreatic cancer				
Locally advanced	reference			
Metastatic	1.83 (1.12–2.99)	0.016	1.73 (1.01–2.96)	0.046
Firstline Chemotherapy regimen				
5-FU	reference			
Etoposide + cisplatin	-			
FOLFIRINOX	0.22 (0.02–3.02)	0.258		
Gemcitabine	1.74 (0.23–13.57)	0.580		
Gemcitabine + Cisplatin	0.25 (0.01–5.01)	0.364		
Gemcitabine + Nab-paclitaxel	2.41 (0.32–18.18)	0.394		
TS-1	0.67 (0.06–8.25)	0.758		
Firstline Chemotherapy dose				
Full dose	reference			
Adjust dose	1.38 (0.45–2.56)	0.305		
Firstline Chemotherapy response *				
PR	reference			
SD	1.18 (0.54–2.58)	0.680		
PD	7.30 (1.40–37.94)	0.018		

BMI, body mass index; ECOG, Eastern Cooperative Oncology Group; 5-FU, 5-fluorouracil; FOLFIRINOX, combination regimen of 5-fluorouracil, leucovorin, irinotecan, and oxaliplatin; TS-1, Tegafur, Gimeracil, and Oteracil combined oral chemotherapy drug; PR, partial response; SD, stable disease; PD, progression disease; * Chemotherapy response evaluation was assessed every 2–3 months using CT and graded according to the Response Evaluation Criteria in Solid Tumors, version 1.1.

**Table 5:** Adverse events of chemotherapy.

	Adverse Events	
	Grade 1 or 2 No. of Patients (%)	Grade 3 or 4 No. of Patients (%)
Hematologic		
Neutropenia	4 (8.5)	0 (0.0)
Febrile-neutropenia	0 (0.0)	1 (2.1)
Thrombocytopenia	1 (2.1)	0 (0.0)
Nonhematologic		
Fatigue	22 (46.8)	6 (12.8)
Anorexia	8 (17.0)	0 (0.0)
Vomiting	1 (2.1)	1 (2.1)
Diarrhea	0 (0.0)	2 (4.3)
Constipation	3 (6.4)	0 (0.0)
Sensory neuropathy	2 (4.3)	0 (0.0)
Mucositis	1 (2.1)	0 (0.0)

## Data Availability

The data that support the findings of this study are available from the corresponding author upon reasonable request.
